# Magnetic resonance imaging-based approaches for detecting the efficacy of combining therapy following VEGFR-2 and PD-1 blockade in a colon cancer model

**DOI:** 10.1186/s12967-024-04975-5

**Published:** 2024-02-23

**Authors:** Xi Xu, Mengjie Ma, Kunlin Ye, Dong Zhang, Xinhui Chen, Jiayang Wu, Xukai Mo, Zeyu Xiao, Changzheng Shi, Liangping Luo

**Affiliations:** 1 Medical Imaging Center, The First Affiliated Hospital of Jinan University, Guangzhou, 510630 China; 2 Department of Radiology, The Second Affiliated Hospital, School of Medicine, South China University of Technology, Guangzhou, 510080 China; 3 The Guangzhou Key Laboratory of Molecular and Functional Imaging for Clinical Translation, Jinan University, Guangzhou, 510632 China

**Keywords:** IVIM-DWI, BOLD-MRI, Immune checkpoint inhibitors, Angiogenesis inhibitors, Vascular normalization

## Abstract

**Background:**

Angiogenesis inhibitors have been identified to improve the efficacy of immunotherapy in recent studies. However, the delayed therapeutic effect of immunotherapy poses challenges in treatment planning. Therefore, this study aims to explore the potential of non-invasive imaging techniques, specifically intravoxel-incoherent-motion diffusion-weighted imaging (IVIM-DWI) and blood oxygenation level-dependent magnetic resonance imaging (BOLD-MRI), in detecting the anti-tumor response to the combination therapy involving immune checkpoint blockade therapy and anti-angiogenesis therapy in a tumor-bearing animal model.

**Methods:**

The C57BL/6 mice were implanted with murine MC-38 cells to establish colon cancer xenograft model, and randomly divided into the control group, anti-PD-1 therapy group, and combination therapy group (VEGFR-2 inhibitor combined with anti-PD-1 antibody treatment). All mice were imaged before and, on the 3rd, 6th, 9th, and 12th day after administration, and pathological examinations were conducted at the same time points.

**Results:**

The combination therapy group effectively suppressed tumor growth, exhibiting a significantly higher tumor inhibition rate of 69.96% compared to the anti-PD-1 group (56.71%). The f value and D* value of IVIM-DWI exhibit advantages in reflecting tumor angiogenesis. The D* value showed the highest correlation with CD31 (r = 0.702, *P* = 0.001), and the f value demonstrated the closest correlation with vessel maturity (r = 0.693, *P* = 0.001). While the BOLD-MRI parameter, R2* value, shows the highest correlation with Hif-1α(r = 0.778, *P* < 0.001), indicating the capability of BOLD-MRI to evaluate tumor hypoxia. In addition, the D value of IVIM-DWI is closely related to tumor cell proliferation, apoptosis, and infiltration of lymphocytes. The D value was highly correlated with Ki-67 (r = − 0.792, *P* < 0.001), TUNEL (r = 0.910, *P* < 0.001) and CD8a (r = 0.918, *P* < 0.001).

**Conclusions:**

The combination of VEGFR-2 inhibitors with PD-1 immunotherapy shows a synergistic anti-tumor effect on the mouse colon cancer model. IVIM-DWI and BOLD-MRI are expected to be used as non-invasive approaches to provide imaging-based evidence for tumor response detection and efficacy evaluation.

## Background

Colorectal cancer is one of the most deadly cancers [1], and about 50% of patients were diagnosed at advanced stages, which faced poor prognosis and high mortality risk. The 5 year survival rate of patients with advanced stage colorectal cancer is approximately 12% [2]. Chemotherapy combined with targeted drugs has played a pivotal role in patients with advanced cancers. However, a fraction of patients display acquired resistance and side effects to the treatment caused by long-term use of drugs. Over the last decade, immunotherapy has emerged as a remarkable success in several refractory cancers [3]. In particular immune checkpoint inhibitors of cytotoxic T lymphocyte antigen-4 (CTLA-4) and programmed cell-death receptor 1 (PD-1)/programmed cell-death ligand 1 (PD-L1) have been approved for various types of metastatic cancers [4]. Immune checkpoint is a negative regulatory molecule that inhibits the excessive activation of immunoreaction, while immune checkpoint inhibitors block this negative regulatory mechanism to increase anti-tumor immune response [5]. Despite the inspiring positive clinical results of immunotherapies for several types of late-stage cancers, only about 10 ~ 35% of patients achieved durable responses among patients treated with single agents, even with these immunotherapy advances [6, 7]. The current challenge is to enhance anti-tumor immunity and improve the objective response rates of patients.

It’s shown that the aberrant structure and function of tumor vasculature may influence tumor behavior and cause therapeutic refractoriness [8]. Insufficient perfusion and dysregulation of vascular adhesion molecules caused by abnormal vessels in tumors may lead to reduced lymphocyte infiltration, ultimately creating immune suppression. A recent study indicated that increased intratumor perfusion could improve the infiltration and activation of lymphocytes, and enhance the efficacy of immunotherapy [9]. Thus, there is a critical need for combination therapies of immune checkpoint blockade therapy and anti-angiogenesis therapy. Hodi et al. [10] reported that the combination of anti-angiogenesis and CTLA-4 blockade substantially improved the disease-control rate (67.4%) and extended median survival time (25.1 months) in patients with metastatic melanoma. Similarly, Allen Elizabeth et al. [11] found that anti-angiogenesis can synergistically promote the effects of anti PD-L1 immunotherapy by enhancing anti-tumor immunity of the tumor microenvironment. It has also been reported that the combination of PD-L1 blockade and vascular endothelial growth factor receptor 2 (VEGFR-2) inhibitor in part of solid tumors showed good safety and durable clinical benefits [12]. However, Yuhui Huang et al. [13, 14] pointed out that anti-VEGFR2 antibody treatment can promote vascular normalization and enhance the efficacy of anti-cancer agents in a dose-dependent and time-dependent manner. Yet, high-dose anti-angiogenesis therapy may lead to significant anti-vascular effects and hinder drug uptake. Therefore, optimizing the treatment plans of combination therapy plays a crucial role in improving the anti-tumor effect. It is quite important to establish biomarker to predict the tumor vascular response, especially the balance between anti-vascular effects and vascular normalization.

Over the past few decades, MRI has provided excellent soft tissue imaging and has been extensively utilized for cancer diagnosis and treatment assessment [15]. Sorensen AG et al. [16] found that the perfusion-related parameter K^trans^ of dynamic contrast-enhanced MRI (DCE-MRI) could reflect the hemodynamics changes during anti-angiogenesis therapy, which closely correlated with overall survival rate and progression-free survival rate in recurrent glioblastoma patients. Another study on the feasibility of IVIM-DWI related perfusion parameters (D* and f) in monitoring the vascular normalization window, and revealed that the parameters of IVIM-DWI showed good correlations with DCE-MRI [17]. In contrast to cytotoxic therapy, which can often be evaluated based on the tumor size reduction, the assessment of treatment response in immunotherapy is frequently characterized by a delayed manifestation [18]. Based on preliminary imaging research results on blood perfusion, it is critically important to use non-invasive real-time imaging approaches to evaluate tumor response in vivo, such as the tumor hemodynamics and tumor hypoxia status during combination treatment. Thus, we intent to explore meaningful imaging indicators for tumor response detection and efficacy evaluation, and provide imaging clues for the formulation of optimal individualized treatment plans.

## Materials and methods

All animal experiments were approved by the Institutional Animal Ethics Committee of Jinan University and strictly conducted in accordance with Institutional Laboratory Animal Care and Use Manual.

### Cell culture and animal model

The murine MC-38 cells were obtained from the Pharmaceutical College of Jinan University and cultured in high-glucose DMEM containing 10% fetal bovine serum (FBS) and 1% penicillin/streptomycin (P/S) at 37 ℃, 5% CO_2_.

A total of 60 male C57BL/6 mice (6–8 weeks of age, 15–25 g of body weight), were purchased from Beijing Vital River Laboratory Animal Technology Corporation (Beijing, China) and raised in a specific pathogen-free condition. The mice were subcutaneously injected with MC-38 cells (0.2 ml of 1 × 10^6^/ml) into the right flank near the hind limbs to develop colon cancer xenografts. We initiated our experiments after the mean tumor volume reached about 150–200 mm^3^ [volume = (length × width^2^) × π/6], which was sufficient for the growth of tumor with relatively high vascularity and absence of obvious necrosis, allowing evaluation of treatment effects and tumor microenvironment changes by function MRI.

We selected 48 mice with colon cancer xenograft tumors for the experiment. Eighteen mice were randomly allocated to three groups: group A (n = 6) received non-specific rat IgG (5 mg/kg, Bio-X Cell) administration as control group; Group B (n = 6) received anti-mouse PD-1 blocking mAb alone (5 mg/kg, catalog BE0273, clone: 29F.1A12, Bio-X Cell) administration; Group C (n = 6) received VEGFR-2 monoclonal antibody (5 mg/kg, Clone: DC101, Bio-X Cell) and anti-PD-1 antibody (5 mg/kg) treatments, and anti-PD-1 antibody was injected 1 h after DC101 administration to avoid drug interaction. All mice were administrated via intraperitoneal injection at 3-d interval and underwent T1WI, T2WI, IVIM-MRI and BOLD-MRI scans before and on the 3rd, 6th, and 9th day after administration. We attempt to monitor the cell proliferation and apoptosis, intratumor perfusion, tumor angiogenesis, hypoxia status and T cell infiltration, during antiangiogenic treatments combined with immunotherapy according to the MRI parameters and pathological findings.

### MRI examinations

All MRI scans were conducted on GE Discovery MR750 3.0 T System (GE Medical System), equipped with a human eight-channel HD wrist coil. The imaging mice were anesthetized by administering with intraperitoneal injection of 0.1% pentobarbital before scanning. T1-weighted images (T1WI) were detected using fast spin-echo (FSE) sequences: repetition time (TR) = 400 ms, echo time (TE) = 11.5 ms, slice thickness = 2 mm, (field of view) FOV = 10 × 10 cm^2^, matrix size = 384 × 224, number of excitations (NEX) = 2. T2-weighted images (T2WI) were acquired using fast recovery fast spin-echo sequences: TR = 3463 ms, TE = 78 ms, slice thickness = 2 mm, FOV = 10 × 10 cm^2^, matrix size = 384 × 288, NEX = 2. BOLD-MRI was conducted with a 3D spoiled gradient echo sequence: eight TEs (TE = 4.9, 9.7, 14.4, 19.2, 23.9, 28.6, 33.4, 38.1), TR = 500 ms, FOV = 10 × 10 cm, slice thickness = 2.0 mm, matrix size = 160 × 160 and NEX = 2. IVIM-DWI MRI was acquired using a free-breathing, single-shot, echo-planar imaging pulse sequence: TR = 3000 ms, TE = 102.4 ms, slice thickness = 2.0 mm, matrix size = 196 × 196, and FOV = 10 × 10 cm^2^. We applied diffusion gradients in three orthogonal directions with 13 b values (b = 0, 25, 50, 75, 100, 150, 200, 400, 600, 800, 1000, 1200, 1500 s/mm^2^).

### Image analysis

The quantitative analysis of MR data was conducted in a consistent manner by two senior radiologists on the dedicated post-processing workstation of Advantage workstation Version 4.5 (AW4.5, GE Healthcare). The BOLD-MRI data were analyzed by the Functool-R2Star program to obtain the transverse relaxation rate (R2*). The R2* maps for each tumor were reconstructed by linearly fitting a single exponential model of the ln (signal intensity) to TE curve. The slope of ln(signal intensity) versus TE determines the R2* (l/T2*) value [19]. We analyzed the IVIM-DWI data by the Functool MADC software. The biexponential model of IVIM-DWI was expressed as SI/SI_0_ = (1–f) × exp(-bD) + f × exp(-bD*), where SI_0_ refers to the mean signal intensity of the region of interest (ROI) at b = 0 s/mm^2^, and SI refers to the signal intensity at higher b values. D is the true diffusion coefficient (which is also called the water molecule diffusion), D* represents the pseudo-diffusion coefficient (referring to microcirculation perfusion), and f represents the perfusion fraction. We referenced the T2w images to detect the changes in tumor volume, and manually drew the region of interest (ROI) by outlining the tumor border at the largest cross-section to obtain each quantitative parameter.

### Histological and immunohistochemical analyses

Thirty of 48 tumor-bearing mice were exclusively set for pathological analyses. Three mice without any treatment were divided as the baseline group (day 0). Twenty-seven mice were randomly divided into three groups, and received the same intervention as MRI subgroups. Three mice from each administration subgroup were randomly selected and sacrificed on days 3, 6, and 9 for histologic analyses. All the mice (n = 18) underwent MRI examinations were sacrificed after the last scanning and regarded as the pathological results of day 12. The excised tissues were immersed in a 4% paraformaldehyde solution, subsequently embedded in paraffin, sliced to a thickness of 5 μm, and then stained with hematoxylin and eosin (HE) following the standard procedures. Staining for Ki67 to assess the proliferative capacity of tumor cells using an anti-Ki67 antibody (1:1000; Servicebio, China), and terminal-deoxynucleoitidyl transferase mediated dUTP nick end labeling (TUNEL) immunofluorescent staining was performed to assess apoptotic cells using an TUNEL kit (Servicebio, China) according to the manufacturer’s instruction. Staining hypoxia-inducible factor-1alpha (HIF-1α) to assess tumor hypoxia using a monoclonal anti-HIF-1α antibody (1:100; Servicebio, China). Staining for the endothelial marker CD31 to measure microvascular density using an anti-CD31 antibody (1:1000; Servicebio, China). The α-smooth muscle actin (α-SMA) (1:1000; Servicebio, China) staining was performed to assess vessel maturity. The vessel maturity index (VMI) was defined as the ratio of positive α-SMA to CD31 staining. CD8a (1:600; Servicebio, China) staining was utilized to assess the infiltration of CD8a within the tumor.

All the sections were observed using an Olympus BX 53 microscope, and three relatively representative fields were selected for photography in each staining of the sections. Three typical fields were chosen for each section, and the average value of the three selected fields is considered the final value. Image-Pro Plus 6.0 software (Media Cybernetics, MD, USA) was used to analyze the positive staining rate of different antibodies.

### Statistical analysis

The statistical analysis in this study was conducted using SPSS 13.0 software (IBM Corporation, Chicago, IL, USA), and statistical plot line charts were generated using GraphPad Prism 6.0 software (GraphPad Software Inc., San Diego, CA). The Kolmogorov–Smirnov test was employed to assess the normal distribution of quantitative data. The tumor volume, imaging parameters and pathological indicators among different time points in each group were compared using a one-way analysis of variance (ANOVA) with least significant difference (LSD) as a post hoc test. At the observation endpoint (day 12) within the three groups, Pearson correlation analysis was used to analyze the correlation between MRI quantitative parameters and pathological indicators. A *P* value < 0.05 was considered statistically significant. An r ≥ 0.8 was considered to be a very strong correlation, whereas 0.6–0.79 be strong, 0.4–0.59 be moderate, 0.2–0.39 be weak, and 0–0.19 is considered to be a very weak correlation [20].

## Results

### The anti-tumor effects of each group on tumor growth

**Fig. 1:**
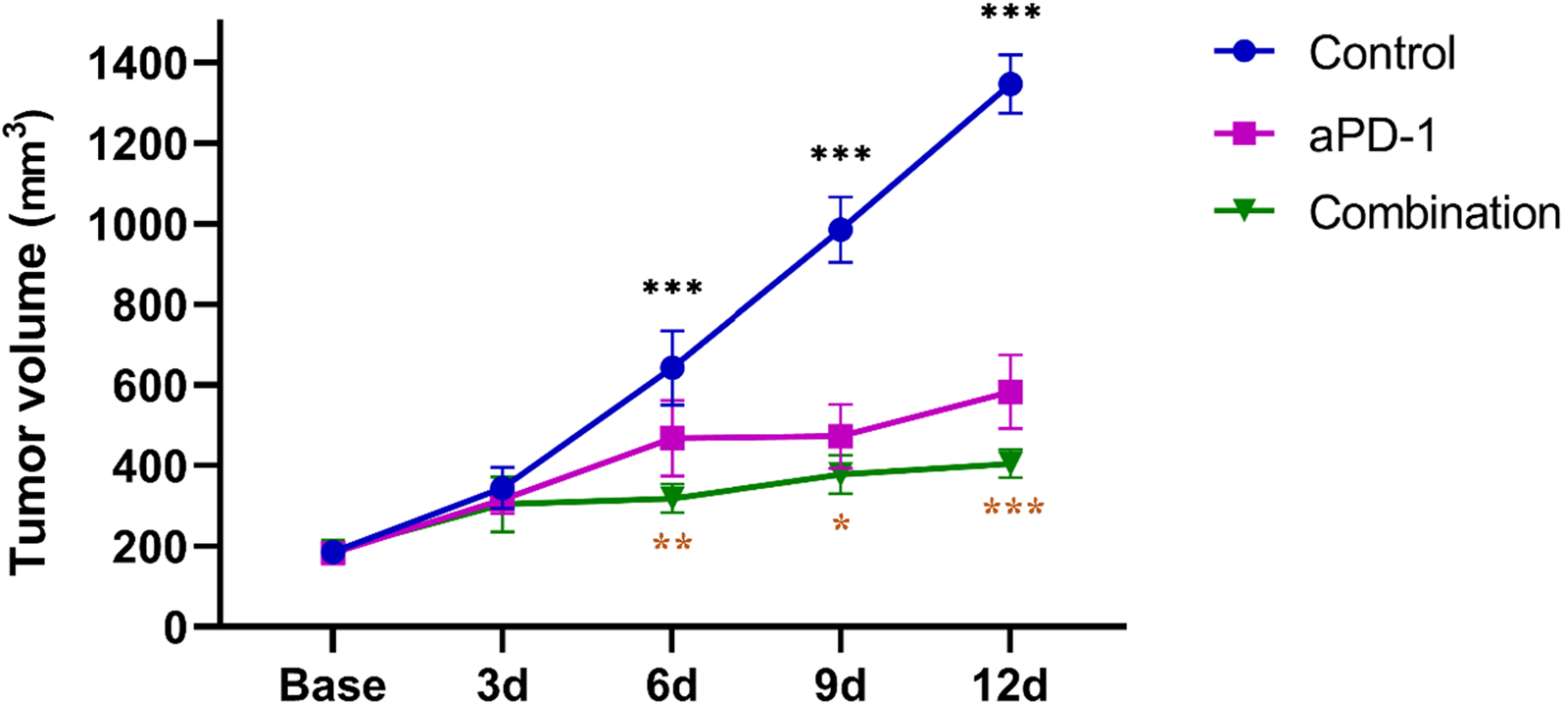
Longitudinal monitoring of tumor growth at different time points in each group, and the data are presented as the mean ± standard deviation. The tumor volumes among different time points in each group were compared using a one-way ANOVA test (*P < 0.05, **P < 0.01, ***P < 0.001). If the test was significant, LSD test was performed to test further significant differences between variables. We marked the time points where significant differences existed between the combination therapy group and anti-PD-1 therapy  group (*P* < 0.05, *P* < 0.01, *P* < 0.001)

The tumor growth at different time points after administration showed that (Fig. 1, Table 1), the combination therapy group inhibited tumor growth from the 6th day after treatment. At the final observation time point (12th day), the combination therapy group showed the smallest tumor volume, followed by the anti-PD-1 treatment group and the control group, with statistically significant differences (F = 338.346, *P* < 0.001). The combination therapy group demonstrated a higher tumor inhibition rate of 69.96%, compared to the anti-PD-1 group (56.71%). From the tumor growth curve, it can be inferred that the combination therapy enhanced the anti-tumor effect superior to monotherapy alone.

**Table 1:** The tumor volumes at each time point in each group (mean ± standard deviation, mm^3^)

	Control	aPD-1	Combination	F	*P*	*P*^*a*^		
						Control vs aPD-1	Control vs combination	aPD-1 vs combination
Base	185.800 ± 20.093	183.650 ± 17.526	189.000 ± 21.387	0.112	0.895	NS	NS	NS
3d	344.467 ± 48.244	315.767 ± 33.637	303.867 ± 65.036	1.020	0.384	NS	NS	NS
6d	642.533 ± 87.318	468.350 ± 89.289	318.733 ± 34.216	28.192	< 0.001	0.001	< 0.001	0.003
9d	985.317 ± 77.350	472.850 ± 75.600	378.223 ± 45.691	139.303	< 0.001	< 0.001	< 0.001	0.029
12d	1347.000 ± 69.155	583.100 ± 86.481	404.650 ± 32.692	338.346	< 0.001	< 0.001	< 0.001	< 0.001

The tumor volumes among different time points in each group were compared using a one-way ANOVA test. If the test was significant (*P* < 0.05), LSD test was performed to test further significant differences between variables, and a *P*^*a*^ value < 0.05 was considered statistically significant

*NS* nonsignificant

### MR results

**Fig. 2:**
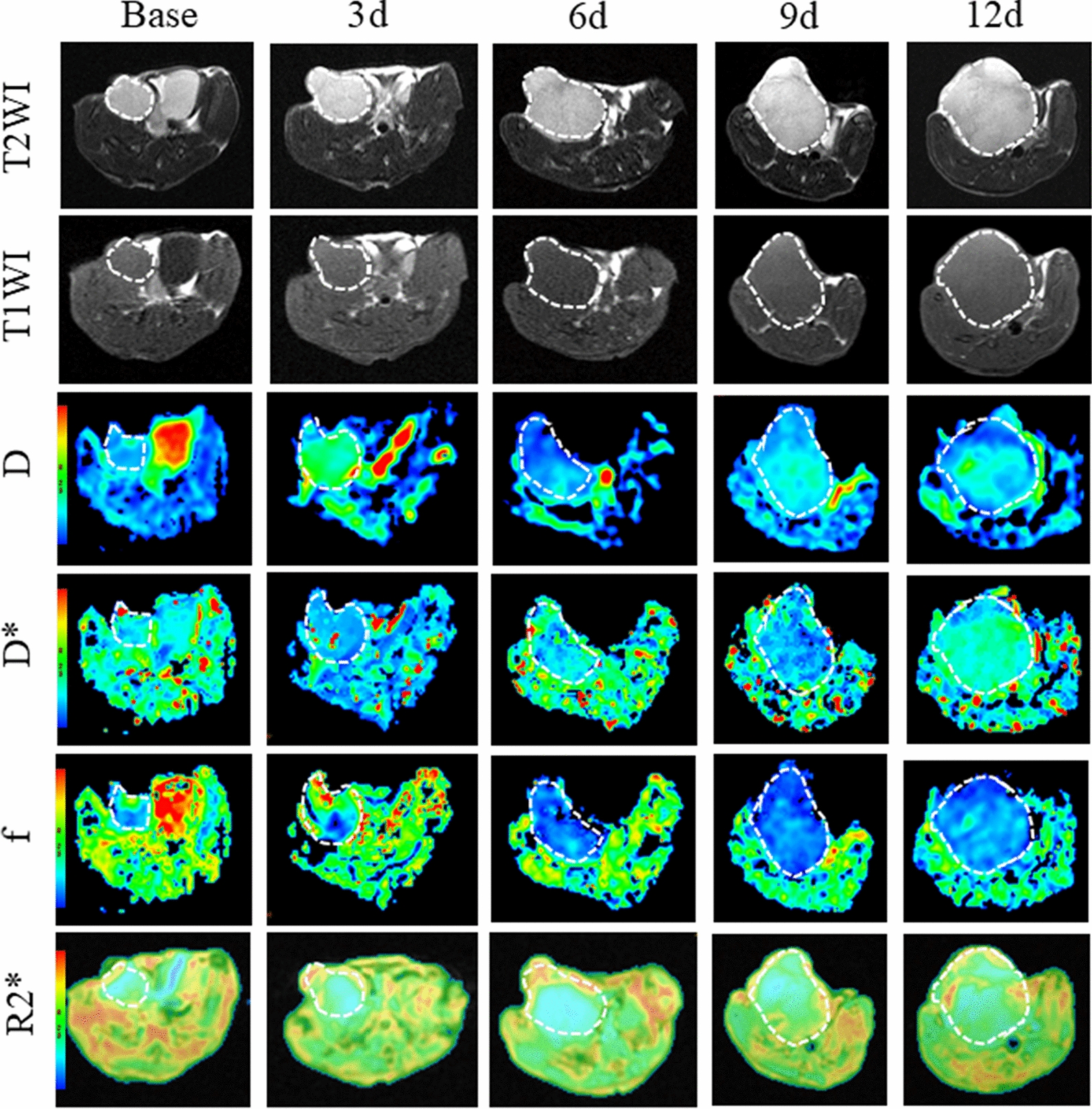
T1 weighted image, T2 weighted image, pseudo-color image of IVIM-DWI (D, D* and f) and BOLD-MRI (R2*) before and after the treatment at different time points of control group. We referenced the T2w image and manually drew the ROI by outlining the tumor border at the largest cross-section to obtain each quantitative parameter

**Fig. 3:**
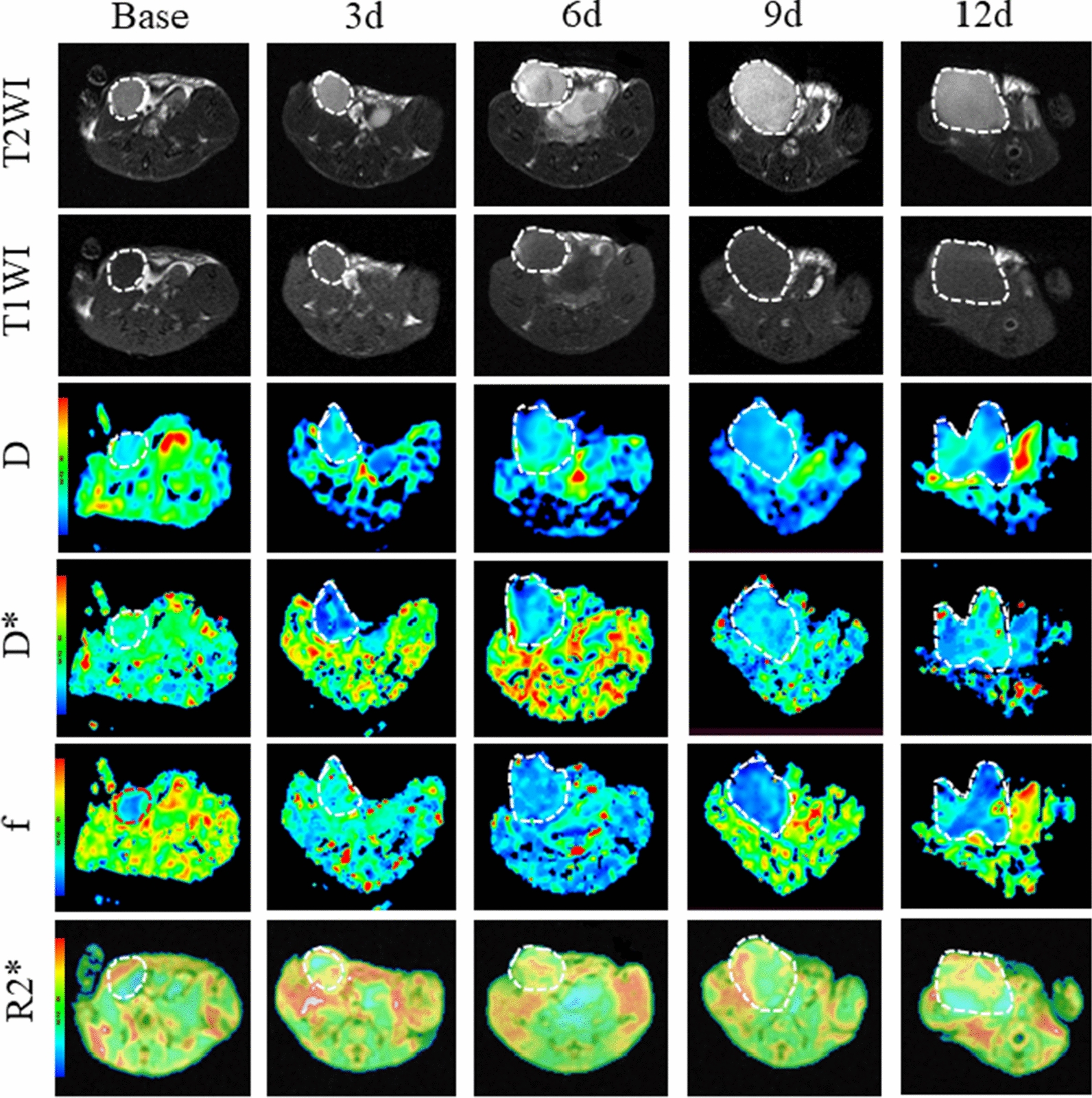
T1 weighted image, T2 weighted image, pseudo-color images of IVIM-DWI (D, D* and f) and BOLD-MRI (R2*) before and after the treatment at different time points of combination group. We referenced the T2w image and manually drew the ROI by outlining the tumor border at the largest cross section to obtain each quantitative parameter

By itself, the tumor volume increased rapidly in the control group. On the 12th day, the tumor showed significant enlargement, appearing as low signal intensity on T1-weighted images and heterogeneous high signal intensity on T2-weighted images (Fig. 2). However, combination therapy significantly delayed tumor growth compared to the control group. On the 6th day, small patchy areas of heightened signal intensity emerged at the periphery of the tumor on T2-weighted images (Fig. 3). Similarly, scattered patch-like areas of slightly high signal intensity could be observed at the tumor periphery on T1-weighted images. It is speculated that these signal changes may be caused by tumor-related hemorrhage.

**Fig. 4:**
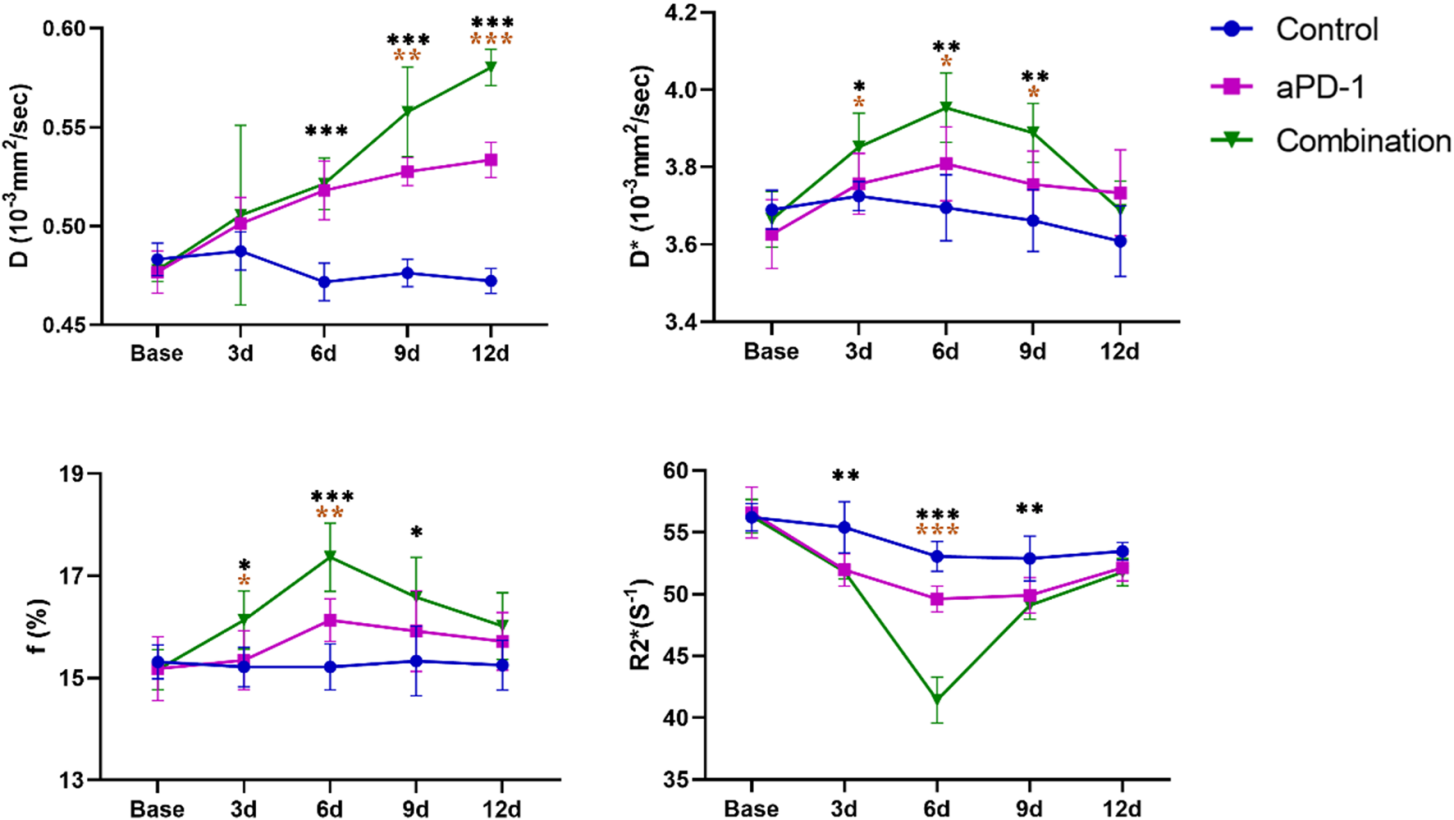
Dynamic changes of IVIM-DWI and BOLD-MRI parameters at different time point in the control group, anti-PD-1 group, and combination group. The data points presented as mean and standard deviation. Comparisons among the three groups at each time point using a one-way ANOVA test (*P < 0.05, **P < 0.01, ***P < 0.001). If the test was significant, LSD test was performed to test further significant differences between variables. We marked the time points where significant differences existed between the combination therapy group and anti-PD-1 therapy group (*P* < 0.05, *P* < 0.01, *P* < 0.001)

Quantitative MRI parameters of this study including D, D*, f, and R2* values exhibited distinct trends among the control group, anti-PD-1 group, and combination therapy group (Table 2, Fig. 4). The D values of the control group showed an overall slight decrease trend (F = 4.251, *P* = 0.009). This could be caused by the increased cellular density during tumor growth, leading to a decrease in the diffusion coefficient of water molecules. The R2* values demonstrated a slight declining trend (F = 6.296, *P* = 0.001), while the overall changes in D * and f values were not significant. In the anti-PD-1 group, the D values exhibited a continuous gradual increase trend (F = 24.876, *P* < 0.001). The f values increased from day 0 to day 6, followed by a gradual decline (F = 2.509, *P* = 0.067). The R2* value decreased gradually until day 6 (F = 22.644, *P* < 0.001). In the combination group, the D values of the combined treatment group significantly increased and reached its peak point on the 12th day (F = 17.534, *P* < 0.001). The D* values and f values showed an initial increase and followed by a decrease after 6 days. The R2* value initially decreased and reached its lowest point on the 6th day, after which it gradually increased (F = 110.054, *P* < 0.001).

**Table 2:** The IVIM-DWI and BOLD-MRI quantitative parameters of the control group, anti-PD-1 group, and combination group in different time points (mean ± standard deviation)

	Base	3d	6d	9d	12d	F	*P*
Control							
D (10^−3^ mm/sec)	0.483 ± 0.008	0.487 ± 0.010	0.472 ± 0.009	0.476 ± 0.007	0.472 ± 0.006	4.251	**0.009**
D*(10^−3^ mm/sec)	3.690 ± 0.051	3.730 ± 0.037	3.700 ± 0.085	3.660 ± 0.080	3.610 ± 0.091	2.246	0.093
f (%)	15.320 ± 0.331	15.220 ± 0.387	15.220 ± 0.449	15.330 ± 0.683	15.250 ± 0.489	0.079	0.988
R2* (S^−1^)	56.221 ± 1.116	55.400 ± 2.074	53.051 ± 1.218	52.891 ± 1.815	53.459 ± 0.723	6.296	**0.001**
aPD-1							
D (10^−3^ mm/sec)	0.477 ± 0.011	0.501 ± 0.013	0.518 ± 0.015	0.528 ± 0.007	0.534 ± 0.009	24.876	** < 0.001**
D* (10^−3^ mm/sec)	3.670 ± 0.072	3.760 ± 0.079	3.810 ± 0.095	3.760 ± 0.086	3.730 ± 0.111	2.010	0.124
f (%)	15.180 ± 0.624	15.350 ± 0.579	16.130 ± 0.418	15.920 ± 0.791	15.720 ± 0.564	2.509	0.067
R2* (S^−1^)	56.589 ± 2.060	51.978 ± 1.317	49.616 ± 1.045	49.914 ± 1.447	52.146 ± 1.065	22.644	** < 0.001**
Combination							
D (10^−3^ mm/sec)	0.478 ± 0.006	0.506 ± 0.045	0.521 ± 0.013	0.558 ± 0.023	0.580 ± 0.009	17.534	** < 0.001**
D* (10^−3^ mm/sec)	3.640 ± 0.092	3.850 ± 0.088	3.950 ± 0.090	3.890 ± 0.076	3.690 ± 0.075	15.090	** < 0.001**
f (%)	15.170 ± 0.393	16.130 ± 0.568	17.370 ± 0.668	16.580 ± 0.778	16.020 ± 0.655	9.951	** < 0.001**
R2* (S^−1^)	56.309 ± 1.368	51.831 ± 0.599	41.436 ± 1.857	49.099 ± 1.139	51.773 ± 1.108	110.054	** < 0.001**

One-way ANOVA test was used to generate the* P*-value. The* P* values in bold indicate statistically significant (*P*<0.05)

### Pathological analysis

**Fig. 5:**
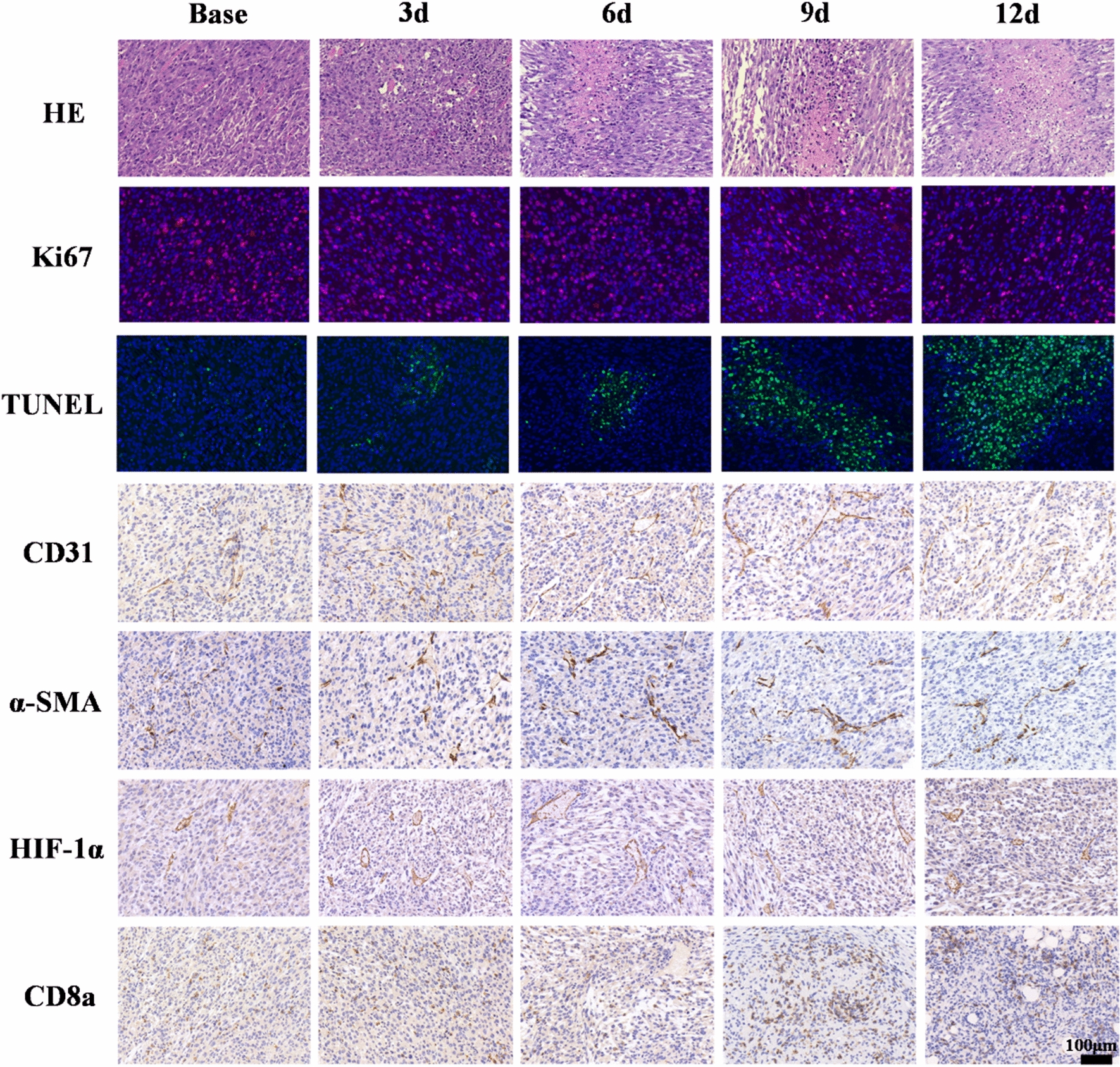
The representative histopathological section of the combination group at different time points, stained with HE, Ki67, TUNEL, CD31, α-SMA, HIF-1α, and CD8a (× 200)

Histological examination using H&E staining demonstrated densely packed and disorganized tumor cells with varying nuclear morphology at baseline (Fig. 5). However, hemorrhage, nuclear condensation, and nuclear fragmentation within the tumor were observed in the early treatment of combination therapy. As time progressed, the necrosis of tumor cells progressively amplified. The positive staining rate of TUNEL (Table 3) in the combination group showed a similar trend to the anti-PD-1 group but with a more significant increase (F = 66.660, *P* < 0.001). The Ki-67 expression in the combination group showed a more noticeable decrease trend (F = 28.409, *P* < 0.001) than anti-PD-1 group. These findings confirmed that the combination therapy exhibited a superior anti-tumor effect compared to the anti-PD-1 group.

**Table 3:** Positive staining rates of pathological indicators in the control group, aPD-1 group, and combination group (mean ± standard deviation)

	Base	3d	6d	9d	12d	F	*P*
Control							
Ki-67 (%)	37.6 ± 1.3	39.9 ± 1.7	42.1 ± 1.4	41.6 ± 1.3	43.3 ± 1.3	7.328	**0.005**
TUNEL (%)	19.2 ± 2.2	18.7 ± 2.7	20.2 ± 2.5	24.4 ± 3.3	24.2 ± 2.9	3.002	0.072
CD31 (%)	35.0 ± 1.7	36.6 ± 1.5	31.9 ± 1.9	30.3 ± 2.3	30.9 ± 2.5	5.673	**0.012**
α-SMA (%)	20.3 ± 2.2	19.8 ± 1.7	15.8 ± 1.5	16.3 ± 1.3	14.1 ± 1.4	7.796	**0.004**
VMI (%)	57.8 ± 3.7	54.2 ± 2.6	49.3 ± 2.0	53.9 ± 5.2	45.6 ± 0.8	6.422	**0.008**
HIF-1a (%)	39.5 ± 1.2	40.3 ± 0.9	38.4 ± 0.9	38.7 ± 1.1	38.5 ± 2.0	1.323	0.327
CD8a (%)	20.5 ± 1.2	21.0 ± 1.4	18.6 ± 1.2	16.8 ± 0.9	12.7 ± 1.7	20.286	** < 0.001**
aPD-1							
Ki-67 (%)	38.4 ± 1.3	37.0 ± 0.3	35.1 ± 1.6	34.4 ± 1.4	31.8 ± 1.7	10.910	**0.001**
TUNEL (%)	18.6 ± 3.1	24.8 ± 2.8	30.7 ± 3.1	42.4 ± 3.2	47.9 ± 4.7	37.609	** < 0.001**
CD31 (%)	35.5 ± 2.5	44.8 ± 2.2	42.4 ± 2.5	32.6 ± 2.5	28.2 ± 4.4	16.535	** < 0.001**
α-SMA (%)	20.6 ± 1.2	25.4 ± 1.8	30.4 ± 2.1	18.7 ± 0.8	15.8 ± 1.7	39.514	** < 0.001**
VMI (%)	58.5 ± 7.2	56.6 ± 2.5	71.6 ± 0.7	57.7 ± 6.3	57.0 ± 10.8	2.823	0.083
HIF-1a (%)	40.5 ± 1.0	42.8 ± 1.0	39.8 ± 1.6	36.8 ± 0.9	36.4 ± 1.1	16.321	** < 0.001**
CD8a (%)	20.7 ± 0.9	22.5 ± 0.7	23.2 ± 0.3	22.8 ± 0.4	17.9 ± 1.1	27.720	** < 0.001**
Combination							
Ki-67 (%)	38.3 ± 1.4	35.6 ± 1.1	34.0 ± 1.5	31.2 ± 1.3	27.7 ± 1.3	28.409	** < 0.001**
TUNEL (%)	19.0 ± 4.3	25.3 ± 4.1	37.0 ± 3.8	52.3 ± 2.9	58.6 ± 2.7	66.660	** < 0.001**
CD31 (%)	33.8 ± 1.9	42.7 ± 2.0	45.6 ± 2.4	37.1 ± 1.9	33.2 ± 2.2	20.695	** < 0.001**
α-SMA (%)	17.6 ± 0.8	30.5 ± 2.6	32.5 ± 1.7	25.1 ± 2.3	18.5 ± 3.0	28.110	** < 0.001**
VMI (%)	52.2 ± 2.5	71.4 ± 3.9	71.5 ± 7.4	67.4 ± 3.3	55.5 ± 7.0	9.245	**0.002**
HIF-1a (%)	40.4 ± 1.3	44.6 ± 0.9	42.6 ± 1.0	36.6 ± 1.4	37.3 ± 0.7	30.085	** < 0.001**
CD8a (%)	20.6 ± 0.7	24.2 ± 0.9	26.0 ± 0.7	25.3 ± 0.9	23.6 ± 1.0	18.188	** < 0.001**

One-way ANOVA test was used to generate the *P*-value. The *P* values in bold indicate statistically significant (*P*<0.05)

**Fig. 6:**
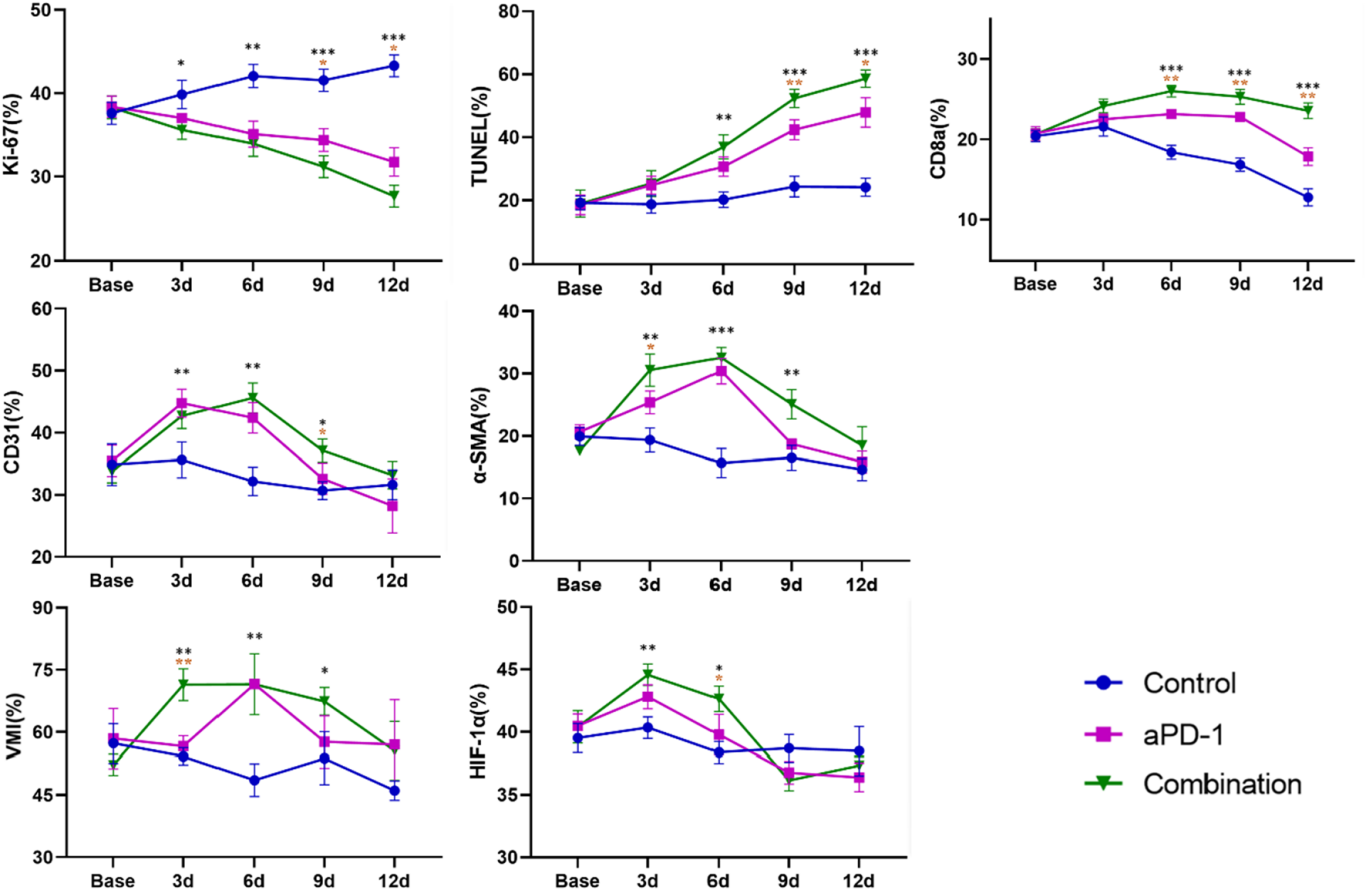
Longitudinal measurements of Positive staining rates of pathological markers Ki67, TUNEL, CD31, α-SMA, HIF-1α, and CD8a in the control, anti-PD-1, and combination groups before and after treatment. Comparisons among the three groups at each time point using a one-way ANOVA test (*P < 0.05, **P < 0.01, ***P < 0.001). If the test was significant, LSD test was performed to test further significant differences between variables. We marked the time points where significant differences existed between the combination therapy group and anti-PD-1 therapy group (*P* < 0.05, *P* < 0.01, *P* < 0.001)

The combination group exhibited an initial increase trend followed by a gradual decline in the positive expression rates of CD31, α-SMA, and VMI (Fig. 6). The VMI in the combination therapy group was significantly higher than the control group on day 3 (F = 28.142, *P* = 0.001), day 6 (F = 25.355, *P* = 0.001), and day 9 (F = 5.698, *P* = 0.041). Additionally, the VMI in the combination group was higher than the anti-PD-1 group on day 3 after treatment, indicating that the combination therapy maintained a high level of vascular maturity over the period. The positive staining rate of HIF-1α in the combination therapy group showed a significant increase on day 3, followed by a gradual decrease until a slight rebound after day 9 (F = 30.085, *P* < 0.001). The CD8a expression in the combination group exhibited an upward trend from day 0 to day 6, followed by a gradual decrease (F = 18.188, *P* < 0.001).

### Correlation analysis

**Fig. 7:**
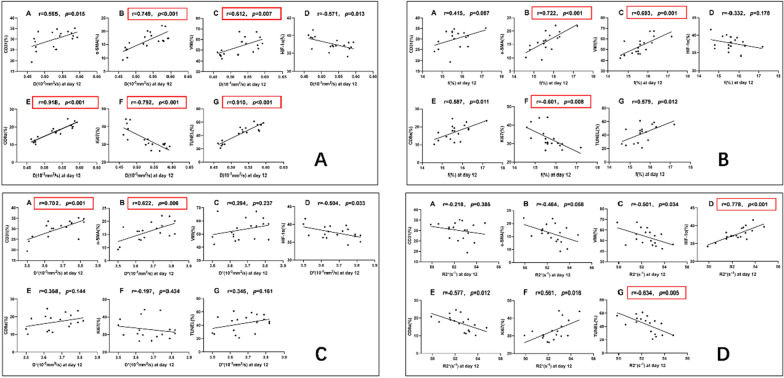
Correlations of IVIM-DWI and BOLD-MRI parameters with pathological indicators. The correlation between the D value (fig. **A**), f value (fig. **B**), D* value (fig. **C**) and R2*value (fig. **D**) with pathological markers Ki67, TUNEL, CD31, α-SMA, HIF-1α, and CD8a, respectively. Quantitative parameters of MRI that exhibit strong or very strong correlations with pathological indicators are highlighted with red boxes

The correlation analysis between the quantitative parameters of MRI and pathological indicators was conducted by Pearson correlation analysis (Fig. 7). In assessing tumor angiogenesis, the D* value showed the highest correlation with CD31 (r = 0.702, *P* = 0.001), the D value exhibited the highest correlation with α-SMA (r = 0.749, *P* < 0.001), and the f value demonstrated the highest correlation with vessel maturity (r = 0.693, *P* = 0.001). In terms of assessing tumor hypoxia, the R2* value was highly correlated with HIF-1α (r = 0.778, *P* < 0.001). When evaluating the infiltration of cytotoxic T lymphocytes into the tumors, the D value showed a significant correlation with CD8a (r = 0.918, *P* < 0.001). In evaluating proliferation activity and apoptosis of tumor cells, the D value was highly correlated with Ki-67 (r = − 0.792, *P* < 0.001), the D value showed a very strong correlation with TUNEL staining (r = 0.910, *P* < 0.001), and the R2* value exhibited a correlation with TUNEL (r = − 0.634, *P* = 0.005).

## Discussion

The malignant tumor is the predominant disease that endangers human health in the present era, and has become a critical health issue worldwide. Improving natural immunity against malignant cells has been a major breakthrough in the treatment of advanced-stage cancer, especially with the successful application of immune checkpoint inhibitors. However, immune checkpoint blockade is only effective for a portion of tumor patients [21, 22]. In recent years, preclinical and clinical studies [11, 23–26] have been carried out on the combination of anti-angiogenic therapy with immunotherapy to enhance tumor response to the treatment. A recent study showed that low doses VEGFR-2 monoclonal antibody can induce vascular normalization, while high doses may cause anti-vascular effects [14]. The key focus of combination therapy is to appropriately promote vascular normalization, reduce vascular permeability, improve effective blood flow perfusion, and increase lymphocyte infiltration within tumor.

In this study, the efficacy of the treatment was dynamically monitored using imaging and pathological examinations, focusing on tumor cell proliferation and apoptosis, intratumor perfusion, tumor angiogenesis, hypoxia status and T cell infiltration. We referenced T2w images to detect the dynamic changes in tumor-bearing mice from the baseline to 12 days after administration. The utilization of MRI enables a visually intuitive measurement of tumor volume, which proves to be more accurate compared to ex vivo measurements using vernier calipers. The results revealed that, the combination group exhibited a higher tumor inhibition rate than anti-PD-1 group from day 6 to day 12. The H&E staining showed marked nuclear condensation and fragmentation within the tumor of the combination therapy cohort on the 12th day, indicating extensive tumor cell necrosis. The expression of Ki-67, a marker primarily indicative of cellular proliferation, displayed a gradual reduction in the combination group. The TUNEL staining, which primarily detects tumor cell apoptosis, exhibited a progressive increase in the combination therapy, surpassing the levels observed in the other groups on the 12th day. Imaging and pathological examinations depicted tumor growth, cellular proliferation, and apoptosis, simultaneously supporting the significantly enhanced therapeutic efficacy of the combination therapy. It’s reported [13, 14] that anti-angiogenesis can promote vascular normalization, accelerate drug uptake, and enhance the efficacy of anti-cancer agents in a dose-dependent and time-dependent manner. Recent studies [11, 24, 27] also indicated that increased intratumor perfusion could improve the infiltration and activation of lymphocytes, stimulate tumor immunity. The combination of the two agents not only encompasses the anti-vascular effect, but also enhances anti-tumor immunity of the tumor microenvironment, and synergistically enhance the efficacy of immunotherapy.

Modifications in the intratumor microenvironment frequently precede morphological alterations in the early stages of treatment. MRI plays a pivotal role in monitoring the tumor immune environment during immunotherapy. Lau et al. [15, 28] used multiparametric MRI to detect the changes in tumor volume (T2-weighted MRI), vascular permeability (Dynamic contrast-enhanced MRI), tumor cellularity (Diffusion-weighted imaging), tumor cellularity and heterogeneity (Diffusion Kurtosis Imaging) in patients with metastatic melanoma receiving immunotherapy. And they described its capability of better predicting short-term and long-term responses to immunotherapy. The recent studies also revealed the potential of MRI to evaluate tumor efficacy during immunotherapy [29–31]. Functional imaging can dynamically evaluate early changes within tumors and predict treatment responses [18]. In this study, we used IVIM-DWI to monitor alterations in water molecule diffusion and perfusion of the tumor-bearing model. D is the true diffusion coefficient (which is also called the water molecule diffusion), D* represents the pseudo-diffusion coefficient (referring to microcirculation perfusion), and f represents the perfusion fraction. The D value showed the most significant increase in the combination therapy group compared to other groups. The D value reflects the diffusion of true water molecules and correlates with cellular density. As the evident suppression of tumor growth, there was a significant reduction in cellular density compared to the control group, resulting in relatively enhanced water molecule diffusion and the most notable increase trend in the D value. The pseudo diffusion coefficient D* value mainly reflects microcirculation perfusion, showed a trend of initially increasing and then decreasing in the combination group, and the D* value was higher than the anti PD-1 group and control group on the 3rd to 9th day after administration. The perfusion fraction (f) values in the combined group exhibited a similar trend, indicating that the combination of antiangiogenic agents and immune checkpoint inhibitors can induce vascular normalization during this time period.

In terms of angiogenesis, the CD31 positive staining rate in the combination therapy group significantly increased on the 3rd to 6th day after administration, and gradually decreased on the 6th to 12th day. CD31, as a platelet endothelial cell adhesion molecule, is a membrane glycoprotein belonging to the immunoglobulin superfamily and expressed in continuous endothelium, but not in discontinuous sinusoidal endothelium [32]. In addition, α-smooth muscle actin (α-SMA) serves as a marker for pericytes in tumor neovessels and reflects vascular maturity. α-SMA staining gradually increased on the 3rd day after administration of DC101, and reached its peak on the 6th day. Similar trends were observed in Vascular Maturation Index (VMI), indicating that vascular inhibitor DC101 increased pericyte coverage, pruned immature vessels, and promoted normalization of vessel structure and function. However, the positive staining rate of the α-SMA staining and VMI of combination group exhibited a higher level compared to the control group from day 3 to day 9, and showed a gradually decreased trend after day 6. Thus, it can be concluded that the combination group induces a transient vascular normalization phenomenon from day 3 to 9 after administration. However, Pan et al. [33] treated the CT26 mouse colon cancer model with the angiogenic inhibitor Endu at a concentration of 5 mg/kg every 2 days. The results revealed that the vascular normalization window was observed between the 4th and 10th day following drug administration. However, Chauhan VP et al. [34] found that the delivery ability of 12 nm nanoparticles through blood vessels enhanced on the 2nd and 5th days during the treatment of 5 mg/kg DC101 every 3 days in E0771 tumor-bearing mice, but returned to baseline levels on the 8th day. It is speculated that the inconsistent normalization window may be due to different types of vascular inhibitors and different duration of experimental observations.

The quantitative parameter R2* derived from BOLD-MRI reflects alterations in deoxyhemoglobin. In this study, it was observed that the R2* values of the combination therapy group and the anti-PD-1 group gradually decreased within the first 6 days after treatment, followed by a rapid rebound after the sixth day. The trend of change in R2* values was more pronounced in the combination therapy group. The initial decline in R2* values during the first 6 days may be attributed to the vascular normalization effect caused by the vascular inhibitor DC101, temporarily improving blood perfusion and alleviating the hypoxic state, resulting in a reduction of deoxyhemoglobin and subsequently lowered R2* values. In the later stage, the vascular inhibitory effect leads to an imbalance in blood supply, causing a gradual recovery of R2* values, which is consistent with the changing pattern of HIF-1α immunohistochemical staining. Similarly, the positive staining rate of CD8a showed initially increased and then decreased in the combination group. This is speculated to be due to a temporary recovery of effective blood perfusion, leading to an increase in infiltrating CD8^+^ T cells within the tumor. As the phenomenon of vascular normalization is transient, and continued treatment will lead to further pruning of tumor vessels and exacerbation of hypoxia. Therefore, a non-invasive real-time imaging approach to evaluate tumor hemodynamics and hypoxia status during combination treatment is critically important.

In this study, it was found that there was a significant negative correlation (r = − 0.792, p < 0.001) between the D value and the Ki-67 positive staining rate, when evaluating cell proliferation activity. In terms of reflecting cell apoptosis, there was a strong positive correlation (r = 0.910, p < 0.001) between D value and TUNEL positive staining rate. Yuan et al. [35] also found a certain correlation (r = − 0.491) between the quantitative parameters of IVIM with Ki67 in a murine rhabdomyosarcoma model. Similarly, they concluded that the D value of IVIM can reflect the proliferation activity in the rhabdomyosarcoma model. This also indicates that the D value has the potential to reflect the proliferation and apoptosis of cells within tumors. In addition, there existed a strong positive correlation (r = 0.778, *P* < 0.001) between the R2* value and the positivity staining rate of HIF-1α, indicating that the R2* value effectively evaluates the hypoxia situation within the tumor. This finding aligns with the study conducted by Robinson et al. [36]. Besides, the pseudo diffusion coefficient D* value and perfusion fraction f value of IVIM-DWI exhibit advantages in reflecting tumor angiogenesis. We also found a good positive correlation between the CD8a positive staining rate and the D value. As the tumor undergoes progressive expansion, the burgeoning density of tumor cells curtails the diffusion of water molecules, resulting in a decrease of the D value and impeding the infiltration of lymphocytes into the tumor. Conversely, a less restricted diffusion of water molecules would foster the infiltration of lymphocytes into the neoplastic site. Therefore, IVIM-DWI and BOLD-MRI demonstrate the ability to provide imaging-based evidence for tumor microenvironment assessment and efficacy evaluation.

There are some limitations in the study. First, this experiment only monitors the therapeutic dynamics in a MC38 subcutaneous-grafted tumor model. Subcutaneous models enhance efficiency and ensure the feasibility and realization of the experiments. However, the controlled environment and uniform tumor growth, while beneficial for reliability, may not fully replicate the complexity of tumor development in their original locations. Despite the limitations, their simplicity allows us to analyze key factors critical to tumor growth and immune responses, providing valuable insights into tumor therapy research. The main focus of our research is to analyze the feasibility of using imaging techniques to monitor tumor perfusion, angiogenesis, and hypoxic conditions. We will validate these conclusions using an orthotopic model in the future experiments. Second, different tumor cell lines exhibit variations in their hypoxic and poorly perfused states, leading to diverse responses to therapeutic agents. Therefore, different tumor cell lines are needed to verify the reliability of these findings. Third, due to the rapid growth of tumors in this study, the scanning time was limited to 12 days. Thus, it is necessary to conduct longer-term longitudinal studies on tumors in situ or transgenic mice in the future.

## Conclusions

The combination of VEGFR-2 inhibitors with PD-1 immunotherapy has been shown to enhance effective blood perfusion, alleviate hypoxia, promote infiltration of CD8a^+^ T lymphocytes into the tumor, and exert synergistic anti-tumor effects on the mouse colon cancer model. The f value and D* value of IVIM-DWI exhibit advantages in reflecting tumor angiogenesis, while the BOLD-MRI parameter, R2* value, is more sensitive in evaluating tumor hypoxia status. In addition, the D value of IVIM-DWI is closely related to tumor cell proliferation, apoptosis, and infiltration of lymphocytes. IVIM-DWI and BOLD-MRI are expected to provide imaging-based evidence for efficacy evaluation and optimization of treatment plans.

## Data Availability

The data of the present study can be made available upon a logical request to the corresponding author and after evaluation of the request by the ethics committee of the university.

## Abbreviations

IVIM-DWI: Intravoxel-incoherent-motion diffusion-weighted imaging
BOLD-MRI: Blood oxygenation level-dependent magnetic resonance imaging
CTLA-4: Cytotoxic T lymphocyte antigen-4
PD-1: Programmed cell-death receptor 1
PD-L1: Programmed cell-death ligand 1
VEGFR-2: Vascular endothelial growth factor receptor 2
DCE-MRI: Dynamic contrast-enhanced magnetic resonance imaging
T1WI: T1-weighted image
T2WI: T2-weighted image
ROI: Region of interest
HE: Hematoxylin and eosin
TUNEL: Terminal-deoxynucleoitidyl transferase mediated dUTP nick end labeling
α-SMA: α-Smooth muscle actin
VMI: Vessel maturity index
HIF-1α: Hypoxia-inducible factor-1alpha
